# Early risk stratification of mortality in the geriatric patients who are at high risk for bleeding and fall from a ground level: an analysis of the national data

**DOI:** 10.5249/jivr.v14i3.1628

**Published:** 2022-07

**Authors:** Nasim Ahmed, Yen-Hong Kuo

**Affiliations:** ^*a*^ Division of Trauma & Surgical Critical Care, Jersey Shore University Medical Center, Neptune NJ USA.; ^*b*^ Hackensack Meridian School of Medicine, Nutley, New Jersey, USA.; ^*c*^ Office of Research Administration, Jersey Shore University Medical Center, Neptune NJ USA.

**Keywords:** Anticoagulation, Coagulopathy, Fall, Geriatric patients, Mortality risk

## Abstract

**Background:**

The purpose of the study is to identify the risk factors of mortality early in patients who have histo-ry of using of anticoagulants or coagulopathy and sustained a ground level fall (GLF).

**Methods:**

The American College of Surgeons Trauma Quality Improvement Program (ACS-TQIP) dataset of the calendar year 2013 through 2016 was accessed for the study. All elderly patients ≥ 65 years old, who were taking an anticoagulant and suffered from a GLF, were included in the study. Other patient characteristics included: sex, race, initial systolic blood pressure (SBP), hypotension (SBP less than 110 mmHg), Injury Severity Score (ISS), Glasgow Coma Scale (GCS) Score, comorbidities such as hypertension (HTN), congestive heart failure (CHF), chronic renal failure (CRF), chronic pulmonary obstructive disease (COPD) and cirrhosis. Multivariable analysis was performed to develop the risk model.

**Results:**

A total of 10,368 patients qualified for the study. Of this total, 788 (7.6%) patients died. The median [IQR] age of the patients was 80 [75-85] years. More than 90% of the patients were white. Fifty-four percent of the patients were female. Approximately 8% of the patients presented with hypotension at the time of hospital arrival. Multivariable analysis showed advanced age, male gender, high ISS, low GCS, presence of hypotension, CHF, CRF, COPD and cirrhosis were highly significant for odds of mortality.

**Conclusions:**

Approximately 8% of the patients, who took an anticoagulant or had a history of coagulopathy and sustained a GLF, died. Certain demographics, higher injury severity and a few comorbidities were highly associated with in-hospital mortality.

## Introduction

Fall is one of the most frequent injury mechanisms occur-ring in the elderly. According to the Centers for Disease Control and Prevention (CDC), 1 in 4 elderly patients each year sustain a fall, which results in numerous injuries and injury-related death and disability.^1-5^ The healthcare cost related to the management of these falls was more than $50 billion in 2015.^6^ Most elderly patients also suffer from many comorbidi-ties that compounds problems after a ground level fall (GLF).^1-7^

Geriatric patients, who sustain a fall and take an anticoagulant or have a history of coagulopathy, have an increased risk of suffering from more severe organ injuries.^8^ Traumatic brain injury is the most common fatal injury identified in these patients.^8^ Given that morbidity and mortality in elderly patients following a GLF is heightened, the CDC developed a set of additional field triage guidelines for Emergency Medical Services (EMS) personnel who encounter elderly patients who have fallen from a ground level position. The guidelines recommend transporting these patients, especially those currently taking anticoagulants to the nearest trauma center.^9^

Prior studies reported the risk of intracranial hemorrhage and associated mortality in elderly patients sustaining a GLF and taking an anticoagulant.^10^ Other studies evaluating the use of anticoagulants and patients falls found variable mortality outcomes. A few studies reported higher mortality as in the previous one, and others reported no significant difference in mortality between patients who were taking anticoagulants or not.^11-13^ However, none of the studies developed a risk estimation at the time of patient arrival to the hospital for in-hospital mortality. Therefore, the purpose of this study is two-fold: 1) identify high-risk patients so that proper resources can be instituted to manage these patients and 2) develop an early risk stratification based on patient characteristics, injury and comorbidities.

## Methods 

The American College of Surgeons Trauma Quality Improvement Program (ACS-TQIP) dataset of the calendar year 2013 through 2016 was accessed for the study. The TQIP dataset is a quality improvement program provided by the ACS. Currently more than 825 trauma centers across the United States participate in the program. The ACS-TQIP provides feedback two times a year to all participating hospitals on outcome measures including in-hospital mortality. All geriatric patients ≥ 65 years old and < 90 years old, who suffered from a GLF and had a history of taking an anticoagulants (warfarin and direct thrombin inhibitors) or coagulopathy, were included in the study. Other patient characteristics included: sex, race, initial systolic blood pressure (SBP), SBP<110 mmHg (hypotension), Injury Severity Score (ISS), Glasgow Coma Scale (GCS) Score, comorbidities such as hypertension (HTN), congestive heart failure (CHF), chronic renal failure (CRF), chronic pulmonary obstructive disease (COPD), and cirrhosis, and chemo-therapeutic medications.

The primary outcome of the study is to evaluate the incidence of mortality and identify the risk factors of mortality early.

***Statistics***

Patient characteristics, injury and outcomes were first summarized as the median with an interquartile range (IQR) [first quartile – third quartile] for continuous variables, and frequency and percentage for categorical variables.^14^ The patients who survived versus who died were compared using Wilcoxon Rank Sum test for continuous variables, and the Chi-square test for categorical variables. Following univariate analysis comparisons, the dataset was split. Eighty (80) percent (n = 8,294) of the patients were randomly drawn without replacement, which constituted the training dataset. The remainder 20% (n = 2,074) of patients constituted the testing dataset for internal validation. 

Multivariable logistic regression analysis was then performed using patient information to obtain a mortality prediction model, which is controlled for age, sex, race, hypotension, ISS category, GCS category and comorbidities (HTN, CHF, CRF, COPD, and cirrhosis) and chemo-therapeutic medications. The model selection process was to find the significant associations between clinical characteristics with in-hospital mortality. The final selection of the model was based on known risk factors and a backward likelihood ratio process as confirmation of factor selection.^14^ A receiver-operating characteristic (ROC) curve, along with a corresponding area under the curve (AUC) calculation and a Hosmer-Lemeshow goodness-of-fit test was used to assess the overall validity of the final model.^14^ Parameter estimates from the fitted model were summarized using β coefficient estimates with 95% confidence intervals, as well as estimated odd ratios (OR) with 95% confidence intervals as measures of precision.^14^ A simple scoring system is created by multiplying the β coefficient of all the significant variables found in the multivariable analysis by factor 10 and rounded to nearest whole number. 

All p-values reported are 2-sided, and a p-value <0.05 is considered statistically significant. Statistical analyses were performed using R statistical software (version 4.0.2).^15^

## Results

***Summary of the patient’s characteristics & Univariate analysis***

A total of 10,368 patients qualified for the study. Out of the total patients, 788 (7.6%) patients died. The remaining 9,580 (92.4%) patients survived the initial hospitalization to the time of discharge from the hospital. The median [IQR] age was 80 [75-85] years. More than 90% of the patients were white. Fifty-four percent of the patients were female. A little more than 8% of the patients presented with hypotension (SBP<110 mmHg) at the time of hospital arrival. When the two groups of patients, who survived and who died, were compared to baseline characteristics, numerous significant differences were found. The patients who died were mostly male (59.1%), and they presented with a higher ISS score and a lower GCS score. Refer to Table 1. 

**Table 1:** Comparison of patients between the groups who survived and who died.

Variable	Values	All Patients (n=10368)	Survived (n=9580)	Died(n=788)	P-Value
**Age**	Median [Q1-Q3]	80 [ 75 - 85 ]	80 [ 75 - 85 ]	81 [ 75 - 85 ]	0.119
**Age Category in years**					0.521
	65-74	2550 (24.6)	2367 (24.7)	183 (23.2)	
	75-79	2118 (20.4)	1961 (20.5)	157 (19.9)	
	80-89	5700 (55)	5252 (54.8)	448 (56.9)	
**Race**					0.346
	American Indian	39 (0.4)	37 (0.4)	2 (0.3)	
	Asian	156 (1.5)	138 (1.4)	18 (2.3)	
	Black or African American	366 (3.5)	344 (3.6)	22 (2.8)	
	Native Hawaiian or Other Pacific Islander	11 (0.1)	11 (0.1)	0 (0)	
	Other Race	297 (2.9)	278 (2.9)	19 (2.4)	
	White	9499 (91.6)	8772 (91.6)	727 (92.3)	
**White**	0	869 (8.4)	808 (8.4)	61 (7.7)	0.543
	1	9499 (91.6)	8772 (91.6)	727 (92.3)	
**Gender**	Female	5600 (54)	5278 (55.1)	322 (40.9)	<0.001
	Male	4768 (46)	4302 (44.9)	466 (59.1)	
**SBP <110 mmHg**	0	9513 (91.8)	8833 (92.2)	680 (86.3)	<0.001
	1	855 (8.2)	747 (7.8)	108 (13.7)	
**SBP mmHg**	Median [Q1-Q3]	146 [ 127 - 165 ]	146 [ 128 - 165 ]	146 [ 123 - 170 ]	0.794
**Pulse rate/minute**	Median [Q1-Q3]	79 [ 69 - 90 ]	78 [ 69 - 90 ]	82 [ 70 - 96 ]	<0.001
**ISS**	Median [Q1-Q3]	10 [ 9 - 16 ]	9 [ 9 - 14 ]	25 [ 10 - 26 ]	<0.001
**ISS Category**					<0.001
	1-9	7690 (74.2)	7413 (77.4)	277 (35.2)	
	10-14	1533 (14.8)	1424 (14.9)	109 (13.8)	
	16-24	1112 (10.7)	723 (7.5)	389 (49.4)	
	25-34	27 (0.3)	16 (0.2)	11 (1.4)	
	35-44	6 (0.1)	4 (0)	2 (0.3)	
**GCS**	Median [Q1-Q3]	15 [ 15 - 15 ]	15 [ 15 - 15 ]	14 [ 4 - 15 ]	<0.001
**Chemotherapeutic agents**	0	10277 (99.1)	9502 (99.2)	775 (98.4)	0.027
	1	91 (0.9)	78 (0.8)	13 (1.6)	
**CHF**	0	8497 (82)	7874 (82.2)	623 (79.1)	0.032
	1	1871 (18)	1706 (17.8)	165 (20.9)	
**Smoking**	0	9748 (94)	8999 (93.9)	749 (95.1)	0.234
	1	620 (6)	581 (6.1)	39 (4.9)	
**CRF**	0	9868 (95.2)	9129 (95.3)	739 (93.8)	0.069
	1	500 (4.8)	451 (4.7)	49 (6.2)	
**CVA**	0	9272 (89.4)	8554 (89.3)	718 (91.1)	0.123
	1	1096 (10.6)	1026 (10.7)	70 (8.9)	
**DM**	0	7341 (70.8)	6779 (70.8)	562 (71.3)	0.772
	1	3027 (29.2)	2801 (29.2)	226 (28.7)	
**History of Angina**	0	10345 (99.8)	9561 (99.8)	784 (99.5)	0.092
	1	23 (0.2)	19 (0.2)	4 (0.5)	
**MI**	0	9936 (95.8)	9188 (95.9)	748 (94.9)	0.216
	1	432 (4.2)	392 (4.1)	40 (5.1)	
**HTN**	0	2612 (25.2)	2368 (24.7)	244 (31)	<0.001
	1	7756 (74.8)	7212 (75.3)	544 (69)	
**COPD**	0	8748 (84.4)	8095 (84.5)	653 (82.9)	0.246
	1	1620 (15.6)	1485 (15.5)	135 (17.1)	
**Cirrhosis**	0	10294 (99.3)	9521 (99.4)	773 (98.1)	<0.001
	1	74 (0.7)	59 (0.6)	15 (1.9)	
**ACS trauma Level**	I	5956 (57.4)	5464 (57)	492 (62.4)	0.004
	II	4412 (42.6)	4116 (43)	296 (37.6)	
GCS= Glasgow Coma Score		ISS= Injury Severity Score		SBP= Systolic blood pressure	
CHF= Congestive heart failure		CRF= Chronic renal failure		CVA= Cerebrovascular accident	
DM= Diabetes Mellitus		MI= myocardial infarction		HTN= hypertension on medication	
COPD= chronic pulmonary disease		N= number of patients		%= percentage	
0= No		1=Yes

$$ Probability of mortality = \frac{EXP(LOG-Odds of mortality)}{1+EXP(LOG-Odds of mortality)} $$

Further analyses of injured body regions, measured by Abbreviated Injury Scale (AIS) score, showed 21.8% of the patients sustained a brain injury. Of the brain injured patients, 66.7% of the patients had a severe head injury with an AIS score ≥3. The associated mortalities for severe and mild to moderate head injuries (AIS score ≤2) was 13% and 8.3%, respectively. The incidence of thoracic and abdominal injuries was 11.2% and 2.5%, respectively. The incidence of pelvic and spinal column injuries was 2.1% and 1.1%, respectively.

***Multivariable analysis and model testing.***

A multivariable logistic regression model was created based on patient characteristics that were present at the time of hospital admission. The model included variables that had a significant difference in the univariate analysis or had a potential correlation with mortality. The variables used in the model were age, sex, race (white vs. nonwhite), ISS, GCS, hypotension, and comorbidities (CHF, CRF, HTN, COPD and cirrhosis). From the regression analysis, the β coefficient provided the estimated change in log odds of mortality for every unit or category change in the predictor variables. Advanced age, male gender, higher ISS, lower GCS, and the presence of hypotension, CHF, CRF, COPD and cirrhosis were highly significant for odds of mortality. Refer to Table 2. 

**Table 2:** Multivariable analysis of risk of mortality.

Variables	β coefficient	OR	95 % CI for OR		p value
(Intercept)	-4.796	-	-	-	<0.001
Age_cat: 2 vs. 1	0.334	1.397	1.029	1.896	0.032
Age_cat: 3 vs. 1	0.613	1.845	1.442	2.379	<0.001
white: 1 vs. 0	0.383	1.467	1.024	2.153	0.043
gender: Male vs. Female	0.464	1.591	1.310	1.935	<0.001
SBP<110 mmHg	0.690	1.993	1.484	2.648	<0.001
ISS_category : 2 vs. 1	0.602	1.825	1.392	2.374	<0.001
ISS_category : 3 vs. 1	1.785	5.959	4.700	7.540	<0.001
ISS_category : 4 vs. 1	2.487	12.024	4.222	32.751	<0.001
ISS_category : 5 vs. 1	3.318	27.612	3.576	170.237	<0.001
GCS_category : 3-8 vs. 13-15	2.992	19.931	14.841	26.893	<0.001
GCS_category : 9-12 vs. 13-15	1.823	6.191	4.190	9.048	<0.001
Chemotherapeutic agent	0.896	2.449	1.092	4.992	0.020
CHF	0.267	1.306	1.026	1.652	0.028
CRF	0.450	1.568	1.046	2.287	0.024
HTN	0.297	1.346	1.088	1.660	0.006
COPD	0.470	1.600	1.242	2.048	<0.001
Cirrhosis	1.447	4.251	2.017	8.346	<0.001
Notes: GCS= Glasgow Coma Score		ISS= Injury Severity Score		SBP= Systolic blood pressure	
CHF= Congestive heart failure		CRF= Chronic renal failure		HTN= hypertension on medication	
COPD= chronic pulmonary disease.		White= (1: yes 0: No)		Age Category= (1: 65-74, 2: 75-79, 3: 80-89)	
ISS Category= (1: 1-9, 2:10-14, 3:16-24, 4:25-34, 5:35-44)			HTN= absence of hypertension was used as reference		

Log-Odds of mortality= -4.796+0.334*Age (if 75-79 years)+0.613*Age (if 80-89 years)+0.383*Race (if white)+0.464*Gender (if Male)+0.69*SBP (if <110 mmHg)+0.602*ISS (if 10-14)+1.785*ISS (if 16-24)+2.487*ISS (if 25-34)+3.318*ISS (if 35-44)+2.992*GCS (if 3-8)+1.823*GCS (if 9-12)+0.896*Chemotherapeutic agent+0.267*CHF+0.45*CRF+0.297*HTN+0.47*COPD+1.447*Cirrhosis

**Figure 1:**
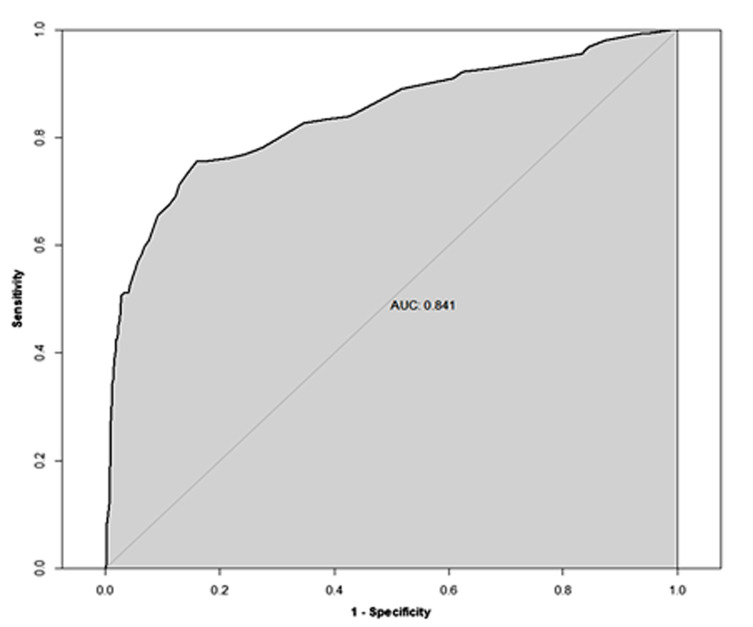
Receiving operating characteristic (ROC) curve for evaluating model performance in predicting mortality based on testing data set.

**Figure 2:**
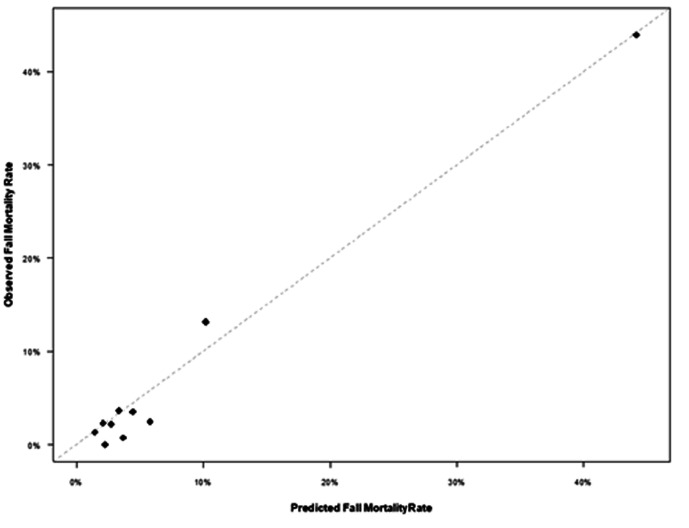
Observe over fitted average mortality in patients who had GLF and on anticoagulation.

In order to check the model for an appropriate fit to predict mortality, a receiver operating characteristic (ROC) curve was created, and an area under the curve (AUC) was generated that showed an AUC of 0.841 [95% CI: 0.808, 0.874]. Refer to Figure 1. In order to validate the results, 20% of the patients were randomly selected to evaluate the observed versus expected mortality. The results showed an adequate comparison. Refer to Figure 2.

The risk of mortality score calculated from the β coefficient ranges from 0 to 76. Refer to Table 3. The higher the score, the higher the mortality. If the score of the patient is 10, the probability of death is 2.2%, that increases to 31% if the score reaches to 40. If the score approaches to 70 the probability of death is 90%. 

**Table 3:** Risk of mortality score.

Variable	Score
Age category: 75-79	3
Age category: 80-89	6
Race (white)	4
Male	5
Hypotension, SBP <110 mmHg	7
ISS: 15-24	6
ISS: 25-34	18
ISS: 35-44	25
ISS: 45-75	33
GCS: 3-8	30
GCS: 9-12	18
Chemotherapy	9
CHF	3
CRF	4
No Hypertension	3
COPD	5
Cirrhosis	14
The scores are based on the estimated coefficient: integer rounding from 10 times the coefficient estimate.	
GCS= Glasgow Coma Score	ISS= Injury Severity Score
SBP= Systolic blood pressure	CHF= Congestive heart failure
CRF= Chronic renal failure	CVA= Cerebrovascular accident
DM= Diabetes Mellitus	MI= myocardial infarction
HTN= hypertension on medication	COPD= chronic pulmonary disease

## Discussion

Approximately 8% of the patients, who took an anticoagulant or had a history of coagulopathy and sustained a GLF, died in the hospital. Advanced age, male gender, higher ISS, lower GCS, and certain comorbidities including, CRF, CHF, COPD and/or cirrhosis were associated with a higher risk of mortality.

As age advances so does the incidence of arterial fibrillation (A-Fib) and use of anticoagulants.^16,17^ The most common reason of the use of anticoagulants in A-Fib is to minimize the incidence of ischemic heart disease and stroke.^18^ Advanced age also leads to an increase in the frequency of falls, fall-related injuries, mortality and morbidities.^5,19^ One of the most common injuries that results in mortality and morbidity is intracranial hemorrhage.^20,21^ Anticoagulants increase the risk of intracranial bleed after a fall and fall-related mortality and morbidity.^22,23,24^ Some risk scoring systems have been developed to apply a balanced approach to elderly patients regarding the use of anticoagulation in A-Fib and the risk of increased bleeding.^10,25^

Geriatric patients who use anticoagulants and experience a GLF are reported to have double the mortality compared to those patients who are not taking anticoagulants.^26^ Studies have shown that brain injury is the single most common injury patients sustain after a GLF, even if a patient’s neurological examination and vital signs are noted to be normal at the scene.^4^ Prior studies have shown the following risk factors associated with the mortality: higher ISS, lower GCS, male gender and comorbidities, including CRF, DM, and HTN.^14^ Since comorbidities play a major role in elderly patient outcomes after a GLF, a study from Spain used prior history of comorbid conditions in their analysis. Approximately 32,000 patients who were ≥65 years old and sustained a fall with a hip fracture were included in the study. The study showed that congestive heart failure had almost 4 times the odds of mortality (OR: 3.88; 95% CI: 3.42-4.41.^27^

Our study evaluated the information of elderly patients who were taking anticoagulation or had a history of coagulopathy and sustained a GLF. Patient characteristics, injury and comorbidities were used. Hypotension was defined as an initial SBP <110 mmHg as described in the recent literature.^28,29^ A risk model was developed to determine the factors associated with in-hospital mortality. As reported elsewhere, the study showed advanced age, a higher injury score, hypotension, CRF, CHF, COPD and cirrhosis are highly significant for hospital mortality.^14,27^ However, our study showed that cirrhosis had the highest correlation with mortality. Furthermore, hypertension and antihypertensive medication compliance had a protective effect on mortality. Our risk model was tested by randomly selecting 20% of the patients. The dataset showed an AUC of 0.84 [95% CI; 0.808. 0.874]. A Hosmer-Lemeshow goodness-of-fit analysis of the risk factors fit the model with p values equaling 0.334, which means the model was a good fit. The expected vs. observed mortality showed most of the decile data concentrated at the diagonal line. 

The information from our study further underscored the value of proper field triage of elderly patients who have a higher tendency of bleeding and coagulopathy. If a patient presents with a SBP <110 mmHg and any comorbidities as above, they should be properly triaged at a trauma center where they can be adequately treated. Our study developed a simple scoring system for the point of care physicians to calculate the predicted mortality very early. Furthermore, if the patient is at a very high risk for mortality after admission to a trauma center, early palliative care consult help facilitate the goals of care. For example, if the patient is a 80-years old, white, male, presented initially with SBP 105 mmHg with GCS score of 8, ISS of 16 and history of CHF and CRF, the probability of mortality is approximately 84 %. That patient should have palliative care consult very early. 

**Limitation**

The study was performed from a large national trauma quality dataset; however, the retrospective nature of the study carries some inherent limitation as any other observational retrospective study. The lack of laboratory data of an anticoagulant effect, type of anticoagulants and detailed information about patient comorbidities may have impacted the results. Furthermore, The TQIP database for these patients has a 90% white majority. This distribution of race may have introduced a racial bias since most trauma patients include a much higher rate of underrepresented minorities.

Conclusion: Elderly patients taking anticoagulants when experiencing a GLF had about an 8% overall in-hospital mortality. Male gender, high severity of injury, hypotension and certain comorbidities were highly associated with mortality. These high-risk patients need extra attention during their hospital stay for the management of injuries, care of their comorbidities. 

**Acknowledgement**

Donald Winters, R.Ph., MPA performed the critical reading and final editing of the manuscript.
